# Artificial intelligence in the healthcare sector: comparison of deep learning networks using chest X-ray images

**DOI:** 10.3389/fpubh.2024.1386110

**Published:** 2024-04-10

**Authors:** M. Akif Yenikaya, Gökhan Kerse, Onur Oktaysoy

**Affiliations:** Faculty of Economics and Administrative Sciences, Department of Management Information Systems, Kafkas University, Kars, Türkiye

**Keywords:** healthcare sector, healthcare organizations, artificial intelligence, deep learning, COVID-19, viral pneumonia

## Abstract

**Purpose:**

Artificial intelligence has led to significant developments in the healthcare sector, as in other sectors and fields. In light of its significance, the present study delves into exploring deep learning, a branch of artificial intelligence.

**Methods:**

In the study, deep learning networks ResNet101, AlexNet, GoogLeNet, and Xception were considered, and it was aimed to determine the success of these networks in disease diagnosis. For this purpose, a dataset of 1,680 chest X-ray images was utilized, consisting of cases of COVID-19, viral pneumonia, and individuals without these diseases. These images were obtained by employing a rotation method to generate replicated data, wherein a split of 70 and 30% was adopted for training and validation, respectively.

**Results:**

The analysis findings revealed that the deep learning networks were successful in classifying COVID-19, Viral Pneumonia, and Normal (disease-free) images. Moreover, an examination of the success levels revealed that the ResNet101 deep learning network was more successful than the others with a 96.32% success rate.

**Conclusion:**

In the study, it was seen that deep learning can be used in disease diagnosis and can help experts in the relevant field, ultimately contributing to healthcare organizations and the practices of country managers.

## 1 Introduction

Globalization has caused significant changes in social dynamics, which in turn culminated in a significant breaking point for sociological balances as well as for human needs and expectations. Factors such as human mobility, population growth, irregular urbanization, changes in eating habits, and climate change have brought along global factors that pose a threat to human health (1). In particular, the surge in the number of high-density cities and the consequent increase in contact areas have led to an increase in the negative effects of global epidemics. In the 20th century, the occurrence of frequent pandemic cases, including the ongoing COVID-19 pandemic that originated toward the end of 2019, has contributed to a heightened comprehension regarding the gravity of global epidemic situations (2).

Epidemic cases affect a wide range of geographical areas and may even have an impact on the whole world. The spread rate of epidemics that can affect mass populations depends on the level of interpersonal contact, the ease of spread of the disease factor and the mode of transmission. In pandemics, large numbers of people being infected at the same time and showing symptoms can cause difficult situations for health institutions. Physicians, who are human beings, becoming ill and switching from being the healthcare service provider to being the receiver (3). This scenario can lead to social chaos alongside loss of life (4). As a matter of fact, this chaos was clearly seen during the flu pandemics of the previous century, namely the Spanish flu (1918), the Asian flu (1957), and the Hong Kong flu (1968). These influenza pandemics caused millions of deaths as well as significant economic, psychological and sociological trauma (5). In addition, due to globalization and sociological changes, there was a noticeable increase in epidemics resulting from respiratory tract diseases as well as the emergence of global epidemics such as SARS, MERS, swine flu, bird flu, zika, and Ebola after the 1990s. These epidemics spread to many countries and thousands of people lost their lives. The most recent epidemic to cause the deaths of millions of people was COVID-19. Even economically developed countries (United States, England, Italy, Spain, Germany, etc.) experienced significant difficulties in combating COVID-19 and incurred significant losses in terms of human lives and the economy (6). The early days of the pandemic particularly involved problems in terms of diagnosis, treatment and medical support, leading to a scenario in which the sector had to decide who would die and who would live due to the lack of human resources and medical supplies (7). Ultimately, leveraging the opportunities presented by the contemporary information age has become imperative in the healthcare sector to mitigate and overcome these detrimental circumstances and scenarios (8, 9). Consequently, the healthcare sector has started to use artificial intelligence (10–12) as a means to address the challenges resulting from the scarcity of qualified human resources and the burden of excessive workload. Therefore, milestones such as the use of vaccines (1796), anesthesia (1846), microscopic organism theory (1861), medical imaging technology (1895), antibiotics (1928), organ transplantation (1954), antiviral treatment technology (1960), stem cell therapy (1970) and immunotherapy (1975) have reached a new milestone with the use of artificial intelligence technologies in this sector (13–16). The present study conducted an empirical examination of the use of deep learning, a type of artificial intelligence, which is widely recognized as a pivotal milestone in the field of healthcare, within the healthcare sector. The study aimed to assess the success of the ResNet101, AlexNet, GoogLeNet, and Xception deep learning networks in detecting COVID-19, viral pneumonia, and disease-free images. Furthermore, if successful, the study aimed to identify the network with the highest success rate.

Pneumonia, whose images were used in the study, is an inflammation caused by organisms such as bacteria and viruses affecting the microscopic air sacs in the lung (17). Approximately 7% of the world population is affected by pneumonia every year and approximately 4 million of the affected patients die due to this disease (18). Typical symptoms of pneumonia, where early diagnosis is extremely important, include shortness of breath, chest pain, severe cough, etc. (19). COVID-19 disease, which was called coronavirus-infected pneumonia in the first periods after its emergence in 2019, can be defined as a virus similar to viral pneumonia, but with more severe symptoms (such as acute respiratory distress, dizziness and severe sweating), with a higher contagion and mortality rate (20). These diseases are generally diagnosed by sputum culture and chest X-ray images (21). In this study, chest X-rays are used for deep learning-based disease diagnosis. Chest X-ray is an imaging technique in which the lung, heart, vascular structures, chest cavity, tissues and bones adjacent to the lung can be examined radiologically with the help of X-ray rays (20). Although X-ray images are very important in terms of disease detection, it can be very time-consuming for doctors to make a diagnosis based on these images (22). Instead, utilizing existing technological possibilities is extremely important in terms of time and cost (20). At this point, chest X-ray images obtained from patients can provide much faster and more successful results compared to existing methods by training deep learning models (23). In this study, this success of deep learning is tried to be determined.

This study has made several contributions to the literature thanks to its subject as well as the findings it wanted to obtain. Firstly, the study includes empirical findings on how artificial intelligence (that is, deep learning, which is a subset) can be used in the healthcare sector, especially in chest X-ray images related to lung diseases, and the extent to which it can reduce the workload of experts in the relevant field. Therefore, the study’s findings demonstrate that deep learning can serve as a viable alternative to mitigate issues such as COVID-19 misdiagnoses associated with RT-PCR and the potential risks faced by doctors in contaminated environments (24) has shown that deep learning can be an alternative method.

Secondly, although there have been studies in the literature addressing the success rate of deep learning networks in detecting COVID-19 (22, 25–27), the number of these studies has remained limited. In addition, the success rates of deep learning networks were not clearly compared in these studies, so the success comparison of the networks in image processing was not determined. In addition, the literature has suggested the use of deep learning models that identify and differentiate between COVID-19 and viral pneumonia (20). In another study, calls were made that lung diseases such as pneumonia, lung cancer and COVID-19 should be examined together (28). In addition, it was stated that the use of artificial intelligence in the health sector is still insufficient due to research inadequacies, so research on this subject should increase (20). Given these circumstances, the study employed the ResNet101, AlexNet, GoogLeNet, and Xception deep learning models, which are widely recognized in the literature and have demonstrated effectiveness in image processing tasks. The present study is the first to compare accuracy rates of these different networks in image detection. Therefore, the findings are expected to help diagnose lung diseases and stop their progression while helping administrators prevent and control diseases. In addition, it is thought that the findings will contribute to the importance of the use of artificial intelligence in healthcare organizations.

## 2 Conceptual framework

### 2.1 Deep learning and its types

Artificial intelligence is a machine or computer application that imitates human-specific features such as reasoning, learning or communicating, and therefore exhibits human-like behaviors (29). This practice is also used to characterize the Fourth Industrial Revolution (30) and improves every aspect of our lives by collecting and learning from data. Used in many activities such as Global Positioning Satellite (GPS), automatic face recognition, image processing, text prediction, financial organization and data management, this practice is more known with its sub-field, deep learning. Because deep learning grows faster than other types of artificial intelligence (30, 31) and can be applied more to different fields of science and business due to its success in discovering complex structures in large amounts of data (24).

Deep learning, which is a part/branch of machine learning-oriented artificial networks (32) is an artificial neural network with multiple layers that allows to extract high-level features from individual data (33). This neural network performs learning using a large amount of data and tries to imitate human behavior (34). Therefore, deep learning is a type of machine learning that uses multi-layered artificial neural networks in fields such as image and speech recognition and grammar processing (35). There are different types in this learning and there are many levels or stages that process data to create a data-based model in these types (36). The types used in deep learning consist of visual geometry group (VGG), AlexNet, ZFNet, GoogLeNet, Xception, Inception, ResNet101, R-CNN, etc. (36–38). Although these network types have a common goal to achieve the highest success in classification, the number of layers differs in terms of filtering, proposed approaches to classification, and the processes followed. This study examines Convolutional Neural Networks (CNN) (24) ResNet101, AlexNet, GoogLeNet, and Xception (38–42), which are relatively more successful in medical image analysis and are commonly examined in the literature. These models are briefly described below.

*Alexnet*: A network of eight learned layers, comprising five convolutional layers and three fully connected layers. This network achieved great success in the classification field in the ImageNet competition held in 2012 where it got its name (43).

*Googlenet*: This network, which was the winner of the ImageNet competition in 2014, consists of 22 layers and the layers are used in parallel, unlike in AlexNet where they are used in order. It makes a difference in terms of calculation cost and memory due to this feature (38).

*ResNet101*: This network has a CNN-based architecture with a depth of 101 layers and is pre-trained with the ImageNet dataset. With this network, objects can be categorized with the fc1000 (1,000 neurons) layer (44).

*Xception*: Xception (45), which develops by building on top of the Inception network and comprises 71 layers, is a network that offers depthwise convolution and pointwise convolution approaches in addition to a normal network that performs operations by moving a filter over multidimensional matrices such as width, height and depth (46).

### 2.2 Studies on deep learning

It is suggested that deep learning, which is one of the sub-branches of artificial intelligence, has allowed for progress in many activities such as navigation, chip design, drug discovery, astrophysics and object recognition, and is therefore widely used in different sectors (32). This machine learning system is also used to great success in the healthcare sector. As a matter of fact, decision-making is facilitated, medical costs and radiological effects are reduced, quality of life is increased, and architectural and technical management can also be improved with activities such as detailed access control and monitoring activity with deep learning practices in the healthcare sector (33). One of the most important contributions of this type of learning to the sector is its success in diagnosing diseases by reducing diagnosis errors, which is one of the sector’s most important problems (47). As a matter of fact, studies in the literature clearly show this success. Coccia (11) suggested that deep learning may be useful in the diagnosis and treatment of epilepsy. Siddiqui et al. (12) found that detecting breast cancer and its stages with the deep learning (IPBCS-DL) model was more successful than current state-of-the-art methods. Shubham et al. (48) used deep learning to identify glomeruli in the human kidney, stating that the accuracy rate of the proposed deep learning model was successful. Deepa et al. (49) used MRI images (normal and tumor) in their study and compared the success of ResNet variants (50, 101, and 152) in detecting the disease. The findings showed that ResNet-152 was more successful in detecting brain tumors. Huong et al. (50) compared the performance of different AlexNet models using skin disease images and determined that AlexNet-SVM was more successful than other models in detecting skin diseases.

Deep learning has also been used in the literature for the detection of pneumonia and COVID-19. Chung et al. (10) attempted to detect COVID-19 before symptoms appeared using deep learning. He successfully developed a deep learning model that diagnoses symptoms using heart rate (HR) data obtained from a smartwatch. Apostolopoulos and Mpesiana (26) have suggested that deep learning models (VGG19, MobileNetv2, Inception, Xception and Inception-ResNetv2) can be used successfully for the detection of this disease in their studies using X-ray images of COVID-19 cases (common bacterial pneumonia, COVID-19, and normal cases). Abd El-Latif and Khalifa (25) examined deep learning (Alexnet, Resnet18, VGG16, and VGG19) and machine learning [Support Vector Machine (SVM), Decision Trees, and Ensemble algorithm] models in COVID-19 x-rays classification. The findings revealed that VGG19 and SVM integration was more successful (98.61%). Pham (22) found in their study using chest X-ray images that some deep learning networks were successful in detecting COVID-19 infections. In their study, Loey et al. (51) created different scenarios (different classes) from X-ray images with four conditions: COVID-19, normal, pneumonia bacterial and pneumonia virus. The Alexnet, Googlenet and Restnet18 deep learning models were used for these scenarios with differing accuracy rates in different scenarios.

The studies mentioned above show that deep learning has beneficial outcomes, especially for the diagnosis and treatment of diseases in the healthcare sector. The findings of this study make it clear that there are different networks (types) of deep learning with successful results for diagnosis and post-diagnosis. However, the success rates of different deep learning networks for each disease were not stated. Although different networks of deep learning have been used for COVID-19 detection (22, 25, 26), there are no studies comparing the success levels between networks for multiple diseases such as COVID-19 and pneumonia. Although these networks of deep learning make successful high-level implications in the relevant field by learning from raw data (for example, images), the processes and approaches to classification differ. This means that the accuracy rates between networks may differ in different diseases. Although there is no clear study addressing the success rates of these networks directly (without creating any scenario) in the detection of COVID-19, different studies have examined this in the healthcare sector. For example, Lee and Nam (52) compared the success rates of AlexNet, GoogLeNet and LASSO for drug response in cancer treatment. The findings revealed that AlexNet and GoogLeNet were more successful than LASSO. Yenikaya and Kerse (38) compared the success rates of AlexNet and GoogLeNet for age-related macular degeneration (AMD) types in the eye. The findings revealed that GoogLeNet had a higher success rate. Khan et al. (42) used deep learning networks to automatically recognize epileptic seizures. The findings indicated that AlexNet had a higher accuracy rate compared to GoogLeNet and SqueezeNet. As seen in all these findings, different deep learning networks may have differing success rates in different types of diseases. This study focused on the classification of COVID-19, viral pneumonia, and disease-free images, employing ResNet101, AlexNet, GoogLeNet, and Xception deep learning networks to assess their respective success rates in this classification task. The study attempted to answer the following questions:

*Research Question 1*: Can deep learning networks (ResNet101, AlexNet, GoogLeNet, and Xception) be successful in detecting COVID, Viral Pneumonia and Healthy X-ray images?

*Research Question 2*: If success is achieved, which network has the highest level of success?

## 3 Materials and methods

**Figure 1:**
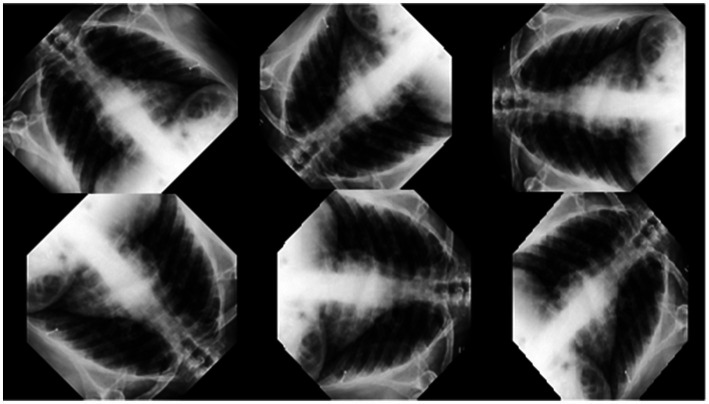
Reproduction of X-ray data.

In this study, the necessary X-ray dataset to detect x-ray images with Covid and Viral Pneumonia and x-ray images without these diseases was obtained from the open access Kaggle website (53). Convolutional Neural Networks were used in accordance with the dataset provided, and the input dimensions were adapted by making changes such as image enhancements. As shown in Figure 1, the input image size was set to 200 × 200 × 3, in a way that allows deep learning models to process data. The images were processed using the 45-degree angle-rotation method in order to tolerate possible error margins and increase the training dataset. A total of 1,680 images reproduced through the method specified from this data set were used, 560 of which are labeled as Covid, 560 of which are labeled as Viral Pneumonia or 560 of which are labeled as Normal X-ray images. A decomposition method was applied at a rate of 70% for the training of the images and 30% for the test (54).

**Figure 2:**
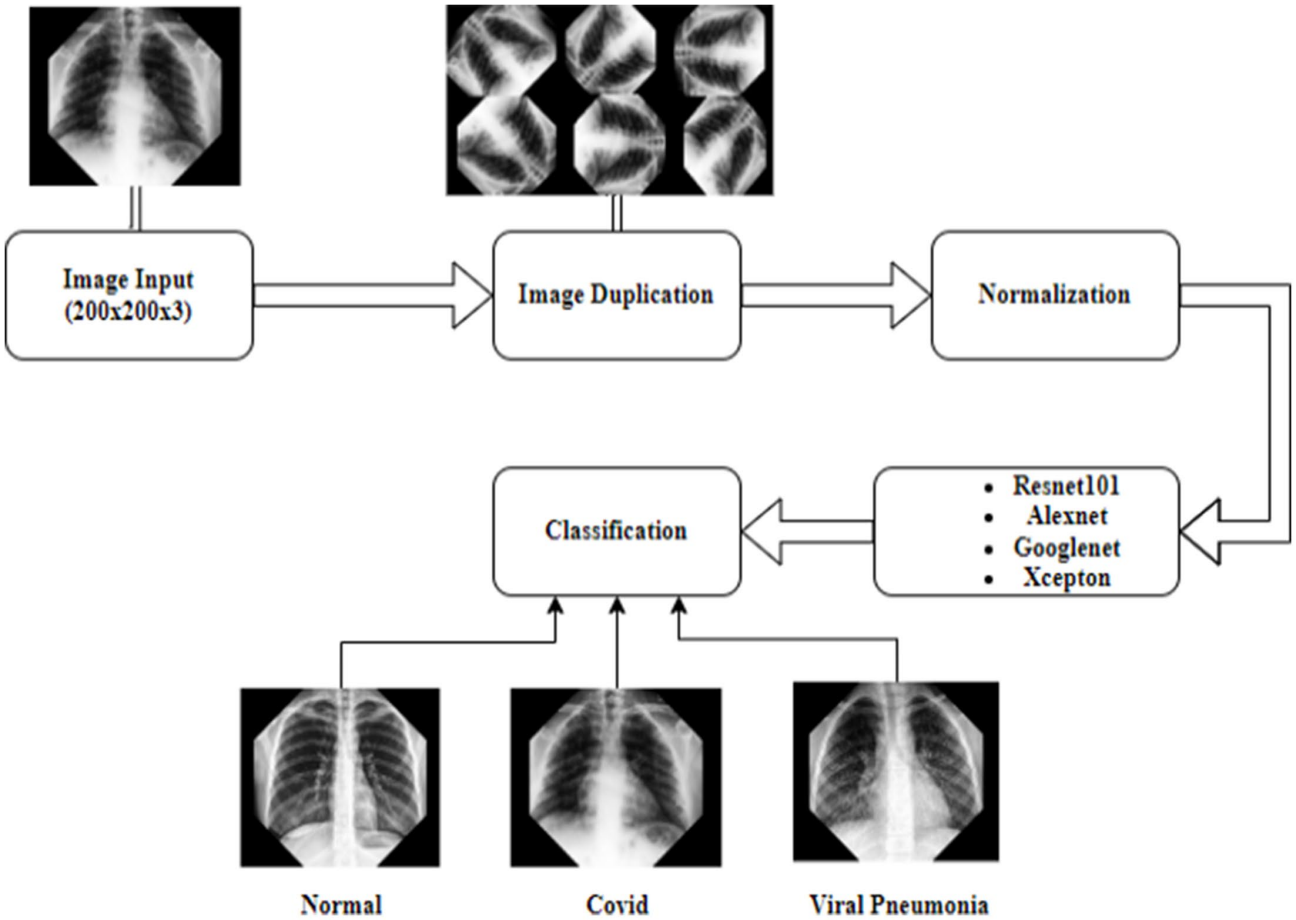
Deep learning flow chart.

Figure 2 shows the flow chart with the process steps of the analyzes made in the research.

## 4 Findings

**Figure 3:**
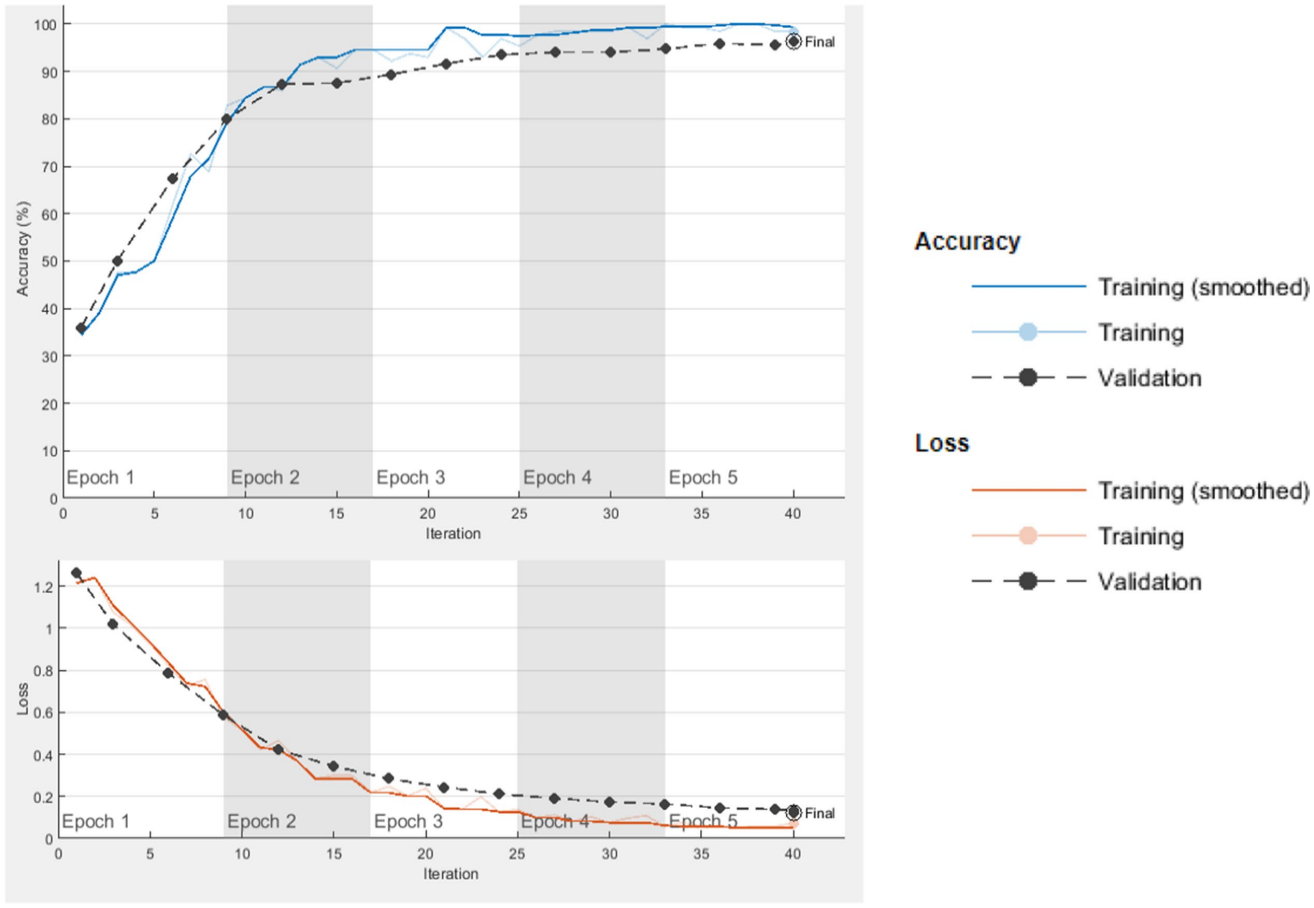
ResNet101 cycle-accuracy and cycle-loss graphs.

**Figure 4:**
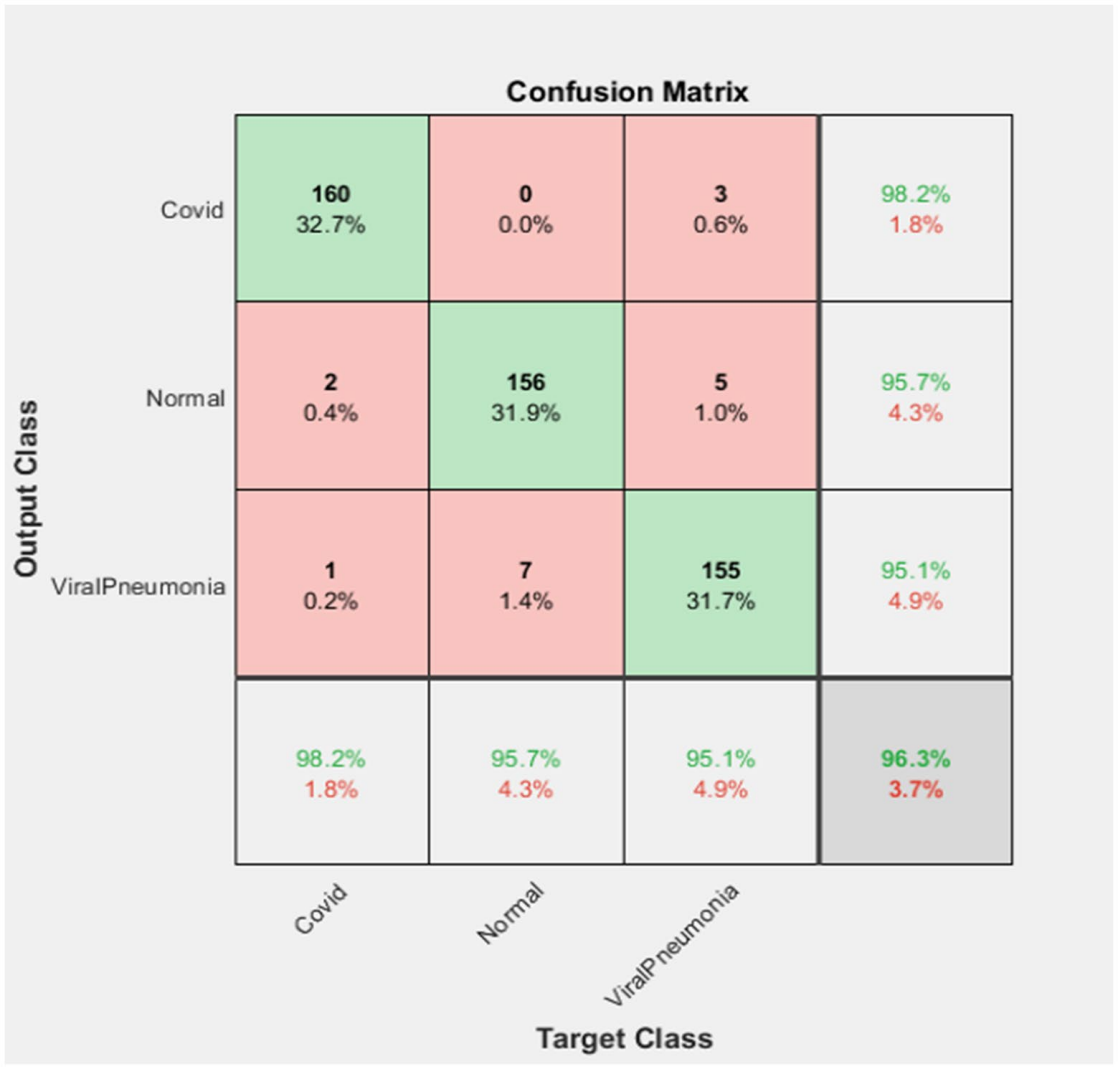
ResNet101 confusion matrix.

The computer used for training in the research has NVidia RTX 4000 Quadro GPU card and 128 GB RAM hardware. Loss functions in the verification dataset were calculated at each step during the training, and a decrease in the loss function value was observed. The training of networks continued at a learning rate of 0.001 for 40 iterations and 5 epochs. As expected, the accuracy rate in all of the analyzes increased with each turnover, and the calculated loss decreased after each turnover. Cycle-accuracy and cycle-loss graphs of the training steps are shown below. Figure 3 shows the graphs of the ResNet101 network while Figure 4 shows the confusion matrix of the same network.

According to the confusion matrix of the ResNet101 deep learning network as stated in Figure 4, 160 of the test data in the set containing 163 Covid images were correctly classified, while 3 of them were identified as Viral Pneumonia. In this case, it was observed that the Covid dataset was classified with a 98.2% success rate.

Again, according to the above figure, 156 of 163 Normal image data were correctly classified. In the Resnet101 network, 2 of them were classified as Covid and 5 of them were classified as Viral Pneumonia. Therefore, it was determined that the normal data set was classified with a success rate of 95.7%.

Finally, 155 of the 163 Viral Pneumonia test data were correctly classified. In this classification, 1 of them was determined as Covid and 7 of them as Normal. In this case, it was observed that the Viral Pneumonia data set was classified with a success rate of 95.1%. In total, it was found that ResNet101 deep learning network achieved a success rate of 96.3%.

**Figure 5:**
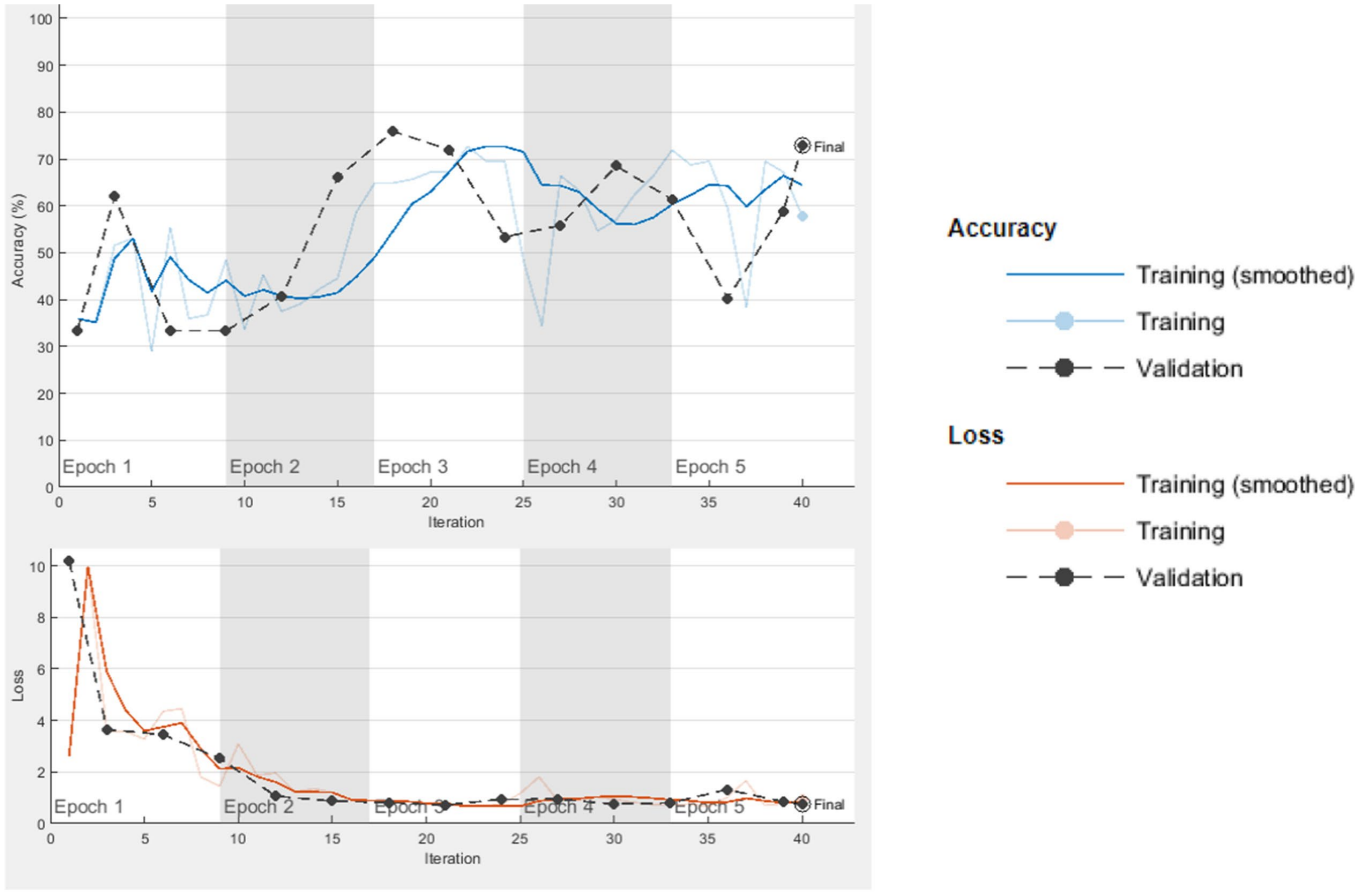
AlexNet cycle-accuracy and cycle-loss graphs.

**Figure 6:**
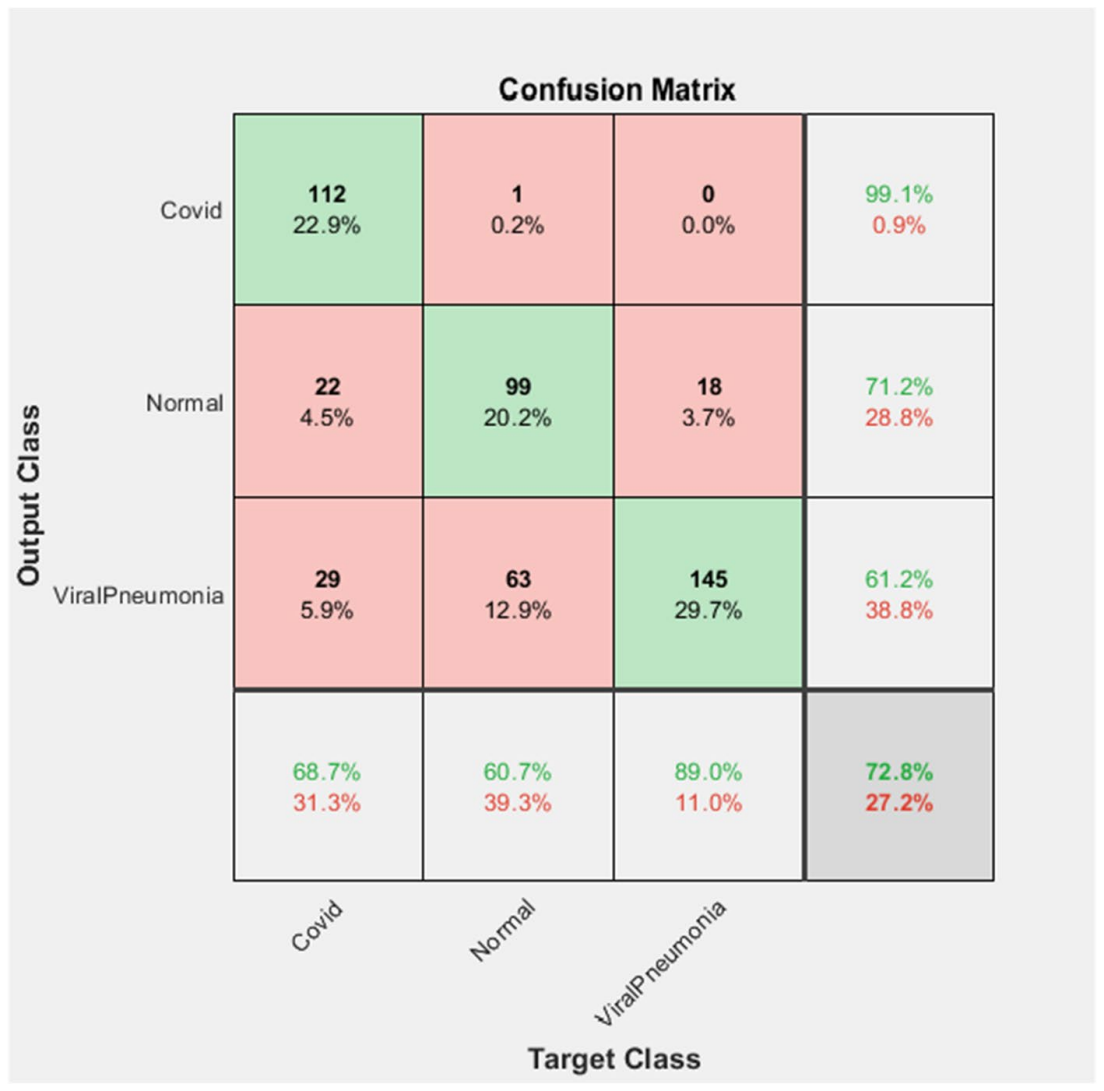
AlexNet confusion matrix.

Figure 5 shows the relevant graphs of the AlexNet network, and Figure 6 shows the confusion matrix of this network.

According to the confusion matrix of the AlexNet deep learning network in Figure 6, 112 of the 113 Covid images in the test dataset were correctly classified as Covid, while 1 of them was classified as Normal. Therefore, the Covid data set classified by AlexNet has a 99.1% success rate.

Again, according to the figure, 99 of 139 Normal image data were correctly classified. 22 of them were classified as Covid and 18 as Viral Pneumonia. In this case, the success rate in the classification of the Normal dataset was 71.2%.

One hundred and forty five of the 237 Viral Pneumonia test data were correctly classified as Viral Pneumonia. Twenty-nine of them were classified as Covid and 63 were classified as Normal. Therefore, the Viral Pneumonia dataset was classified with a success rate of 61.2%. It was found that the AlexNet deep learning network achieved an overall success rate of 72.8%.

**Figure 7:**
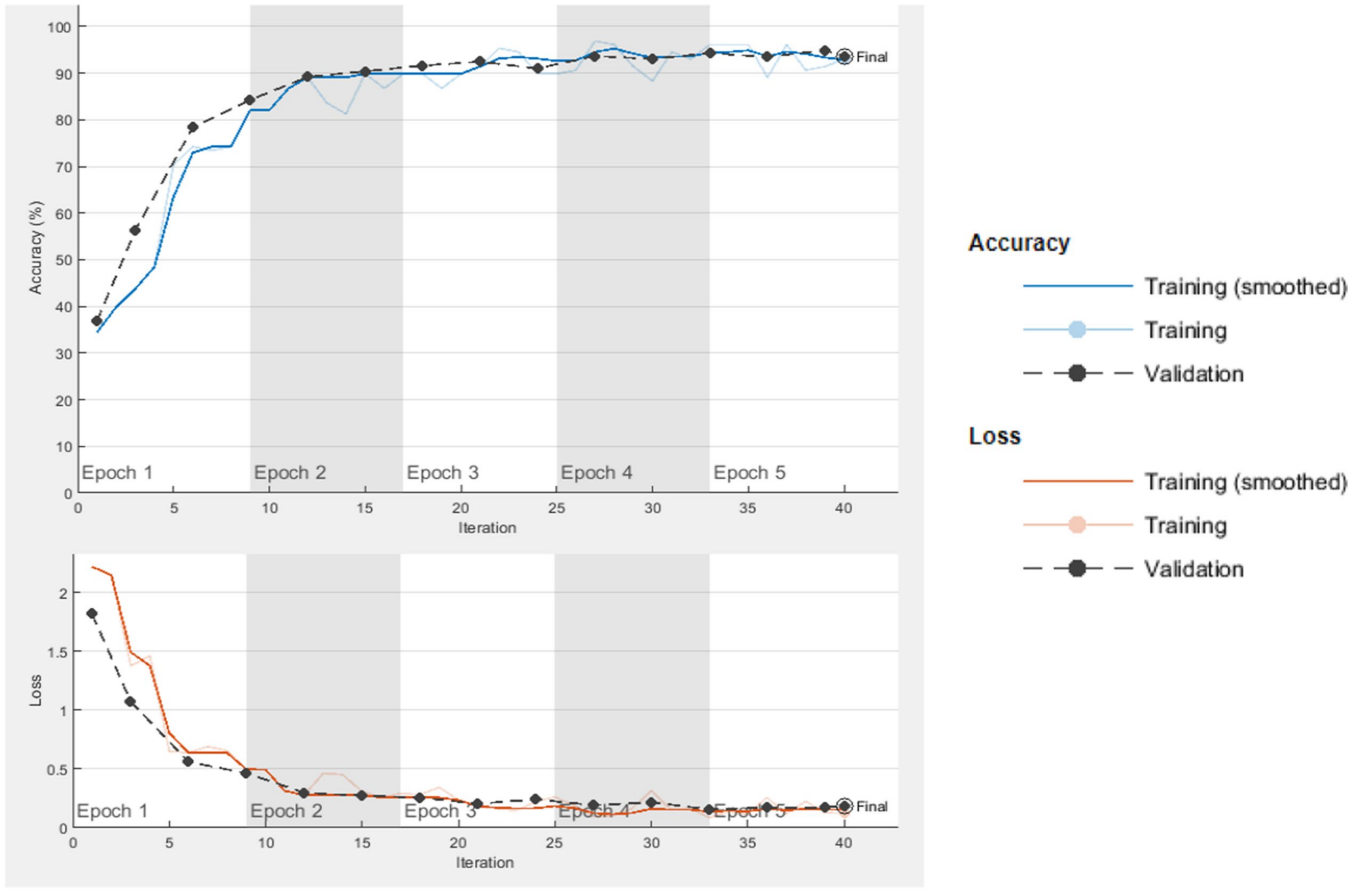
GoogLeNet cycle-accuracy and cycle-loss graphs.

**Figure 8:**
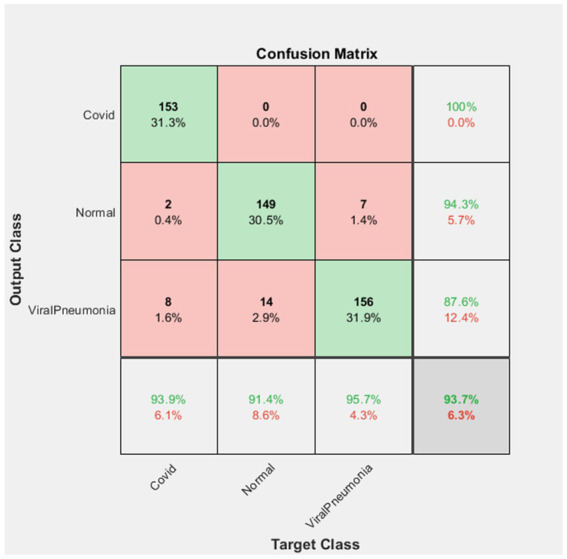
GoogLeNet confusion matrix.

Figure 7 shows the graphs for the GoogLeNet network. In Figure 8, the confusion matrix of this network is presented.

The confusion matrix finding for the GoogLeNet deep learning network stated in Figure 8 shows that all of the 153 test data in the set was correctly classified as Covid. In this case, the Covid dataset were classified with a 100% success rate.

In addition, 149 of the 158 Normal image data were correctly classified. It was observed that 2 of them were classified as Covid and 7 of them were classified as Viral Pneumonia. Therefore, the Normal data were classified with a success rate of 94.3%.

Finally, it was determined that 156 of 178 Viral Pneumonia test dataset were correctly classified while 8 images were classified as Covid, while 14 images were classified as Normal. In this case, the Viral Pneumonia dataset was classified with a success rate of 87.6%. It was found that the GoogLeNet deep learning network achieved a success rate of 93.7% in total.

**Figure 9:**
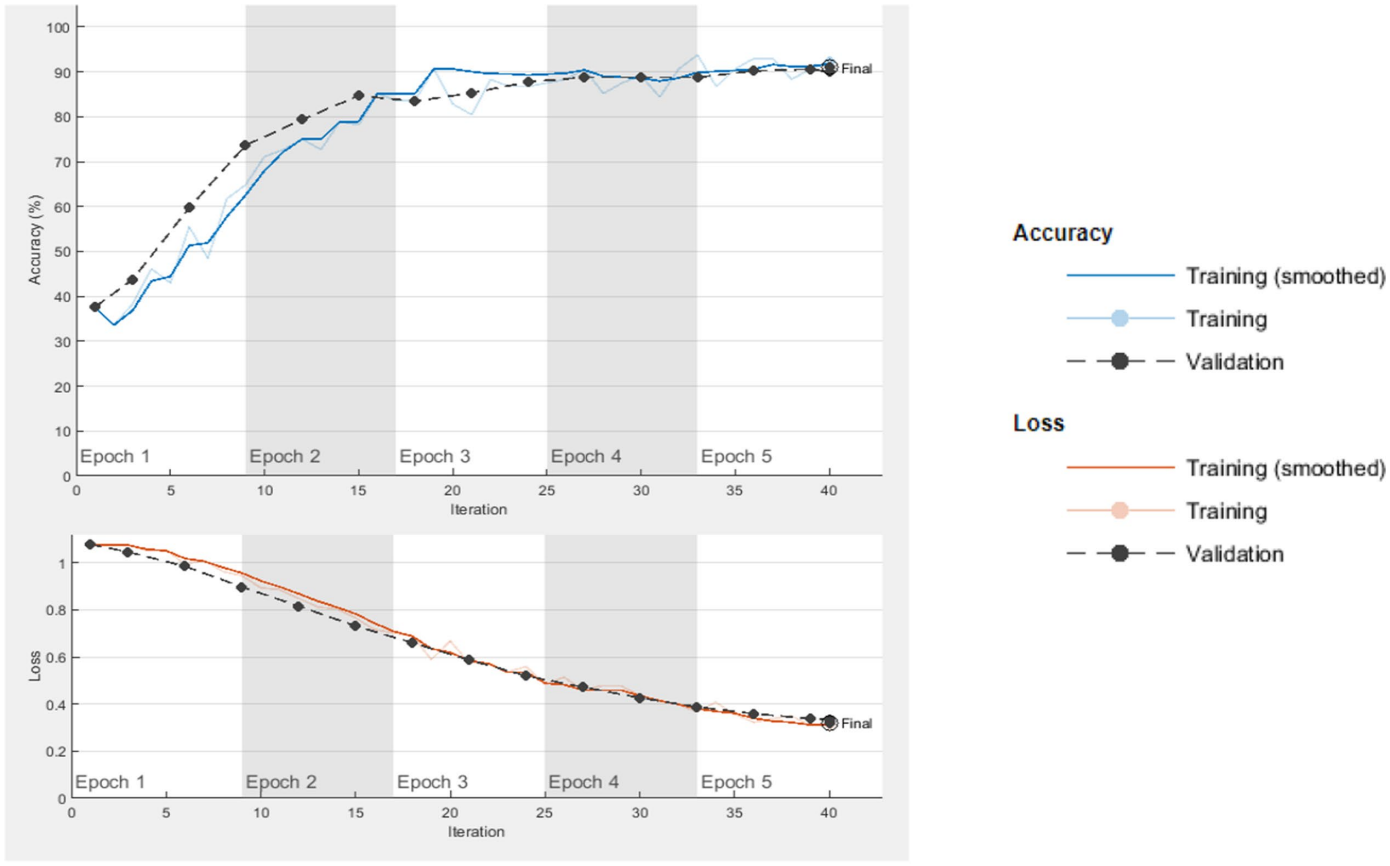
Xception cycle-accuracy and cycle-loss graphs.

**Figure 10:**
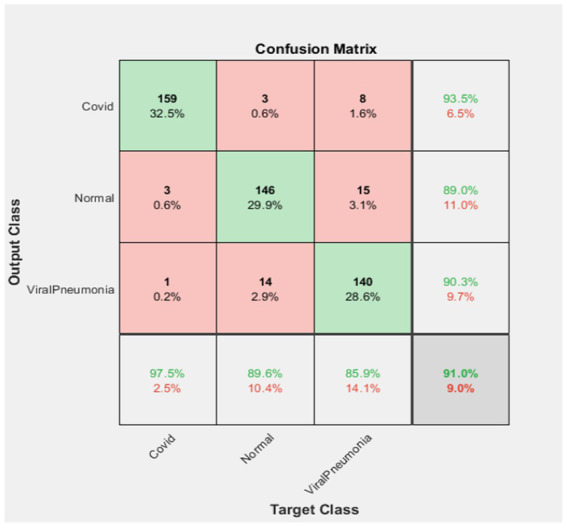
Xception confusion matrix.

Figure 9 shows the relevant graphs of the Xception network, and Figure 10 demonstrates the confusion matrix of it.

According to the confusion matrix of the Xception deep learning network stated in Figure 10, 159 of the 170 Covid images in the test dataset were correctly classified. There are 3 Normal images and 8 Viral Pneumonia images in the dataset classification. Therefore, the Covid dataset was classified with a 93.5% success rate.

Another finding in Figure 10 shows that 146 of the 166 Normal images in the dataset were correctly classified. Three images were classified as Covid and 15 images were classified as Viral Pneumonia. In this case, the Normal dataset has a success rate of 89.0% in classification.

Finally, of the 155 Viral Pneumonia test data, 140 were correctly classified. One image was classified as Covid and 14 images as Normal in this classification. Therefore, the Viral Pneumonia dataset was classified with a 90.3% success rate. Overall, it was found that the Xception deep learning network achieved a success rate of 91.0%.

The accuracy (Equation 1), precision (Equation 2), recall (Equation 3) and F1 score (Equation 4) values of the calculated parameters are given in Table 1. The formulas for these values are presented below.


(1) $$\mathrm{Accuracy}=\frac{TP+TN}{TP+TN+FP+FN}$$

(2) $$\mathrm{Precision}=\frac{TP}{TP+FP}$$

(3) $$\mathrm{Recall}=\frac{TP}{TP+FN}$$

(4) $$F1\;\mathrm{Score}=2*\frac{\mathrm{Precision}*\mathrm{Recall}}{\mathrm{Precision}+\mathrm{Recall}}$$

**Table 1:** Calculated parameters of networks.

Network	Accuracy	Precision	Recall	F1 score
ResNet101	0.9632	1.00	0.9632	0.9632
AlexNet	0.7280	1.00	0.7280	0.7280
GoogLeNet	0.9366	1.00	0.9366	0.9366
Xception	0.9100	1.00	0.9100	0.9100

Values used for important processes, i.e., disease classification, in areas such as machine learning and deep learning include TP (true positive), TN (true negative), FP (false positive) and FN (false negative). However, the accuracy rate alone may not be sufficient because the medical consequences of misclassifications may be different. For example, classifying a data that indicates disease as healthy can have serious consequences. Therefore, parameters such as precision, recall and F1 score should also be considered. The precision value signifies the amount of data that are predicted as positive or diseased and whose actual classification is also positive or diseased. The recall value signifies the amount of data that should be predicted as positive or diseased, whose actual classifications are positive or diseased (55). The F1 score signifies the harmonic average of the precision and recall values. Therefore, the F1 score is usually calculated in cases where FN and FP values are important, and it is used in critical processes such as disease classification (56). These situations were also taken into account, which led to in the findings in Table 1 below.

Considering the findings in Table 1, the highest accuracy rate belongs to the ResNet101 deep learning network with 96.32%. This value was followed by 93.66, 91.00 and 72.80%, respectively. Therefore, in terms of success rate, ResNet101 was followed by GoogLeNet, Xception and AlexNet, respectively.

## 5 Discussion

In today’s world brought about by the information age, digital technologies are developing at a great pace and play an important role in raising life standards. One of the areas where these technologies are used effectively is the healthcare industry. Causes such as increasing workload, qualified human inadequacy, human-induced errors in the diagnosis and treatment stages, long waiting times to use health services, etc. have increased the using rate of digital technologies in a short time. This study examines the deep learning networks used for applying artificial intelligence in the healthcare sector. It aims to determine the success of identifying and diagnosing COVID-19 and Viral Pneumonia, as well as the x-ray images without any diseases, through ResNet101, AlexNet, GoogLeNet, and Xception, which are known as important deep learning networks. Another issue discussed in the context of the research is which networks are more successful than others in terms of separating and identifying of these diseases along with providing reliable outputs. The results and contributions obtained are presented in the following items.

- This study used a total of 1,680 open-source lung x-ray images. 70% of these images, which were grouped as Covid, Viral Pneumonia and Normal (the absence of this disease), were used in training networks, whereas 30% was used in in evaluation. Each network was tested with the same dataset to determine their levels of success. All of the deep learning networks used in the study yielded successful results (with success rates between 73 and 96%) in extracting and diagnosing the data. Therefore, the results have shown that artificial intelligence (that is, deep learning, which is its subset) is a usable tool in the diagnosis of these diseases. The findings are in parallel with the findings of other studies in the literature (22, 25, 26, 51, 57), which suggest that deep learning networks are successful in diagnosing diseases such as COVID-19 and Viral Pneumonia. Therefore, the first of the research questions, *“Can deep learning networks (ResNet101, AlexNet, GoogLeNet and Xception) be successful in detecting COVID, Viral Pneumonia and healthy X-ray images?”* was answered positively.
- In terms of data processing and reaching the result, a comparison was made between the success rates of deep learning networks, which are discussed in the context of the research. It was observed that the highest success rate in identifying the data presented, distinguishing it from other diseases, and making the correct diagnosis belonged to the ResNet101 deep learning network with a success rate of 96.32%. Therefore, the findings answer the question of “*If success is achieved, which network has the highest level of success?*” As mentioned before, different deep learning networks have been used in the literature (22, 25, 58) for the detection of COVID-19, but no comparison has been made for the detection of different diseases using CNNs. In some studies [e.g., (28)], there has been a call to examine lung diseases such as pneumonia, lung cancer and COVID-19 together for future studies. Our study responded to this call (28) with its findings and clearly showed the most successful network (ResNet101) that can be used by comparing the level of success between different networks.
- It can also be said that one of the important contributions of the research compared to the literature is the reproduction of data by the rotation method. In other words, the deep learning networks used in our study were trained with different visual angles. Therefore, the success level of deep learning networks discussed (especially ResNet101) in identifying and diagnosing image data from different angles with this technique may be high.

Global inequality has become more evident than ever in the current time period. The most obvious of these inequalities is undoubtedly the opportunities in the healthcare sector. Criteria such as the health investment rates of countries, the number of physicians *per capita*, the number of deaths due to disease and the level of access to medical needs are some of the factors contributing to this inequality. As a matter of fact, the World Health Organization (WHO) statistics for 2022 show that *per capita* health expenditures are $9,691 in North America and $64 in South Asia. Again, in Africa, 1 doctor serves 3,324 people, while in Europe this number is 293. In addition, one of the important issues emphasized in the same report is a possible “imminent collapse” warning in the health systems of countries (59).

Accordingly, reasons such as the aging world population, the increasing number of chronic diseases, the rise in the average age of physicians, etc. increase the risk of experiencing significant problems at the point of qualified access to health care (60). At this point, considering the success rate of our study, the use of artificial intelligence technologies in the healthcare sector will make significant contributions to reducing the injustice in access to health services and ensuring the sustainability of qualified health services (61). Therefore, Jin et al. (24), artificial intelligence (that is, deep learning, which is a subset of it) can contribute to the healthcare sector with practices such as diagnosing diseases and stopping the progression of the disease, accelerating drug development and improving drug quality, and can help country administrators to take and control measures against diseases.

## 6 Conclusion and recommendation

To draw a conclusion from the findings, it is clearly seen that artificial intelligence can be used effectively for disease diagnosis in the healthcare sector. In this study, all of the deep learning networks considered gave successful results in disease diagnosis, but the Resnet101 network recorded a higher success rate than the others. Therefore, it was determined that this deep learning network can be used in the diagnosis phase. This success of artificial intelligence in disease diagnosis may contribute to healthcare organizations and employees in reducing the workload and shortening the diagnosis process in a COVID-19-like pandemic that may occur in the future. In addition, the use of artificial intelligence in this sector will also help healthcare businesses to achieve their main objectives. Because healthcare organizations, which have the main purpose of meeting the different health services needed by individuals on time, with accurate diagnosis and treatment and at low cost, will be able to achieve this goal with artificial intelligence. Therefore, it will be ensured that both healthcare organizations and healthcare employees are effective and efficient.

This study was carried out specifically for COVID-19 and Viral Pneumonia, and the findings and the success of deep learning networks were presented within these limitations. It is recommended to conduct similar studies on whether different diseases can be detected on x-ray images of lungs. Again, the analyzes in the study were made using a limited amount of data such as 1,680 x-ray images. In future studies, network training and testing with data labeled by more experts in their fields can contribute to increasing the success level of relevant artificial intelligence applications. In addition, as seen in the study findings, ResNet101, which has a higher number of layers, was more successful than other networks. This situation entails the questions of “Does having a high number of layers -especially in research-specific images- increase the level of success?” or “Can the number of layers be kept at the optimum level according to the diseases and the images used as data? In other words, should modifications be made on the layers specific to the network in line with the number of layers and the process?” Therefore, future studies may contribute to the literature and the relevant field by attempting to answer these questions. In addition, it is recommended for future studies to carry out a field application in which traditional methods and artificial intelligence-supported methods will be compared in disease diagnosis. In the case of health enterprises, artificial intelligence can be used in the diagnosis of other diseases due to the lack of qualified human resources and time. For this, it is useful to conduct academic research on the detection of other diseases.

## Data availability statement

Publicly available datasets were analyzed in this study. This data can be found at: https://www.kaggle.com/datasets/pranavraikokte/covid19-image-dataset.

## Author contributions

MY: Writing – review & editing, Writing – original draft. GK: Writing – review & editing, Writing – original draft. OO: Writing – review & editing, Writing – original draft.
